# The least sample size essential for detecting changes in clustering solutions of streaming datasets

**DOI:** 10.1371/journal.pone.0297355

**Published:** 2024-02-20

**Authors:** Muhammad Atif, Muhammad Farooq, Mohammad Abiad, Muhammad Shafiq

**Affiliations:** 1 Department of Statistics, University of Peshawar, Peshawar, Pakistan; 2 College of Business Administration, American University of the Middle East, Egaila, Kuwait; 3 Institute of Numerical Sciences, Kohat University of Science and Technology, Kohat, Pakistan; University of Sindh, PAKISTAN

## Abstract

The clustering analysis approach treats multivariate data tuples as objects and groups them into clusters based on their similarities or dissimilarities within the dataset. However, in modern world, a significant volume of data is continuously generated from diverse sources over time. In these dynamic scenarios, the data is not static but continually evolves. Consequently, the interesting patterns and inherent subgroups within the datasets also change and develop over time. The researchers have paid special attention to monitoring changes in cluster solutions of evolving streams. For this matter, several algorithms have been proposed in the literature. However, to date, no study has examined the effect of variability in cluster sizes on the evolution of cluster solutions. Moreover, no guidance is available on determining the impact of cluster sizes on the type of changes they experience in the streams. In the present simulation study using artificial datasets, the evolution of clusters is examined concerning the variability in cluster sizes. The findings are substantial because tracing and monitoring the changes in clustering solutions have a wide range of applications in every field of research. This study determines the minimum sample size required in the clustering of time-stamped datasets.

## 1 Introduction

The clustering approach is an unsupervised learning problem that considers multivariate data tuples as objects and partitions them into a prespecified number of clusters. Entities in each cluster are comparably similar to one another than entities belonging to different clusters. Commonly, the similarity is measured in terms of a distance function between each pair of objects [1]. Some well-known similarity measures include Euclidean, squared Euclidean, Manhattan, cosine, and Chebyshev distance functions. The notion of a cluster is not specifically defined, and the standards can vary significantly from case to case [2–4]. Some algorithms aim to minimise the intracluster variation, while others identify clusters as the dense region in the feature space. From the perspective of cluster definition, the traditional algorithms are divided into five categories namely partitioning, hierarchical, density-based, grid-based, and model-based clustering algorithms [5].

In today’s world, a bulk of information is continually generated by different sources over time. In these applications, the data are not stationary but rather evolves. Consequently, the interesting patterns and natural subgroups in the datasets do not remain stagnant; they change and evolve over time. In such a dynamic environment, the entire training dataset is not available to the learning algorithm at once. Consequently, a sequence of cluster solutions needs to be generated at some discrete points in time [6]. This phenomenon raises an important question: are these cluster solutions static over time or do they experience any transitions? Significant work has been conducted in monitoring and tracking changes in these clustering solutions obtained at successive time points in all these years.

Over the past two decades, practice of monitoring and tracing the evolution of clustering solutions within data streams has gained considerable importance across various domains. The clusters undergo certain changes, and comprehending the type of transitions that occur can provide a significant advantage. The elements within the clusters may migrate from one cluster to another, leading to the disappearing, merging, and splitting of the clusters at later time points. Similarly, the surviving clusters can adopt internal transitions, such as changes in size, location, and cohesion [7, 8]. Applications of monitoring changes in cluster solutions span a diverse range of fields and scenarios. Some key applications include, businesses utilize cluster monitoring to cluster customers based on purchasing behavior, detecting sudden shifts in cluster solutions can help identify anomalies in data streams, monitoring cluster changes can reveal evolving patterns in patient data, Monitoring cluster evolution in social networks helps uncover changing community structures, tracking changes in species distribution through clustering helps assess ecosystem health and detect potential threats, changes in product data clusters can reveal production variations and help maintain quality standards, etc. Atif et al. [9] provided a comprehensive review of the literature on the various techniques used to monitor and trace the development of clustering solutions over time. The discussion has made it abundantly clear that tracking and monitoring changes in a dynamic environment are essential for forecasting the future and formulating policies. Nevertheless, segmentation studies are inherently exploratory and are greatly influenced by the number of variables and the sample size. A comprehensive simulation study is crucial to estimate the minimum sample size necessary to ensure statistical validity without the need to reduce the number of variables.

The novelty in estimating the minimum sample size necessary for monitoring changes in cluster solutions lies in its pioneering approach. This research breaks new ground by addressing the often-overlooked aspect of sample size determination in the context of evolutionary cluster analysis. By focusing on this critical element, it offers a fresh perspective on ensuring the validity of results, and adapt to evolving data patterns.

The key contribution of the present study is to establish sample size requirements for data-driven segmentation analyses. This provides data analysts with a valuable tool to assess whether the available sample for monitoring and tracking changes in clusters is adequate, considering the number of variables within the segmentation base. It helps analysts determine whether they should collect additional data or reduce the number of variables used in their analysis to ensure robust and meaningful results. A comprehensive review of the literature has been conducted, and the algorithms proposed for tracing the cluster solutions are discussed in the next section.

## 2 Related work

The evolutionary clustering framework was first introduced by Chakrabarti et al. [10]. This framework generate a series of clustering solutions {*ξ*_1_, *ξ*_2_, ⋯, *ξ*_*n*_} at consecutive discrete time points. In other words, it provides a clustering solution *ξ*_*i*_ = {*X*_1_, *X*_2_, ⋯, *X*_*k*_} for each time point *t*_*i*_ in the stream. The algorithm works by simultaneously maximising two criteria. First, each clustering solution must directly represent the dataset at the associated time point as closely as possible. Second, the clustering solution should not significantly deviate from the result obtained at the adjacent time point. The evolutionary clustering framework was initially developed for *k*-means and agglomerative hierarchical clustering techniques. The spectrum clustering problem is expanded within the evolutionary paradigm by Chi et al. [11], producing more reliable and consistent outcomes. Spectrum evolutionary clustering is more stable against long-term drift and less sensitive to short-term noise. However, these algorithms are incapable of detecting the emergence of new clusters and the aging of existing clusters. To address this issue, Zhang et al. [12] combines the concept of evolutionary clustering with density-based algorithms to prevent arbitrary groups from developing and disappearing in dynamic social networks. These techniques greatly improve the evolutionary clustering literature; however, they cannot capture a variable number of clusters over time. The hierarchical Dirichlet process and the hidden Markov model are combined by Xu et al. [13] to handle this problem and significantly boost performance.

Using self-organising maps, Denny and Squire [7] proposed a method for identifying structural changes in cluster solutions of temporal datasets. This approach involved comparing clustering results at various points in time and monitoring how the new results differed from the old one. This research work uses world development indicators to assess the performance of the proposed algorithm. However, this algorithm failed to detect the newly emerged and disappearing clusters. This issue was addressed by the Relative Density Self-Organising Map (ReDSOM), a visualization-based approach developed by Denny et al. [14]. The algorithm could recognize various changes in cluster structures, including the formation of new clusters, disappearance, splitting, merging, expansion, centroid movement, and changes in cohesiveness.

The MONIC framework, used for modeling and monitoring changes in clustering solutions of cumulative data streams over time, was introduced by Spiliopoulou et al. [8]. This framework compares the results obtained at two subsequent time periods to track the structural changes in the clusters. The changes adopted by the clusters can generally be categorized into two groups: external transitions and internal transitions. External transitions encompass the survival, merging, splitting, disappearance, and re-emergence of clusters. Internal transitions involve adjustments in the size, cohesion, and location of the surviving clusters. The overlap, a non-symmetric matrix that serves as the foundation for the MONIC framework, is expressed by the following expression:

$$\begin{array}{c} Overlap\left(X_{i},Y_{j}\right)=\frac{\left|X_{i}⋂Y_{j}\right|}{\left|X_{i}\right|},\;i=1,2,\cdots ,k_{1},\;j=1,2,\cdots ,k_{2} \end{array}$$ (1)

where *X*_*i*_ is a member of the set of clusters produced by the first clustering and *Yj* is a member of the set of clusters produced by the second clustering. A matrix of order *k*_1_*k_2_ is produced, where *k*_1_ and *k*_2_ represent the number of clusters from the first and second clustering, respectively. The similarity index between clusters is represented by the value on the appropriate element of the matrix and acts as a marker for tracking the external transition. The cluster membership is assessed to track the internal transition of the clusters that have survived.

The Monitoring Clusters Transition (MClusT) algorithm, developed by Oliveira and Gama [15], visualizes the transition of clusters on a bipartite graph by utilizing conditional probabilities as edge weights. MClusT incorporates a tracking technique based on graph theory, a transition detection algorithm, and a taxonomy of transitions. For each pair of clusters obtained from the stream at successive time points, the algorithm calculates conditional probabilities. These conditional probabilities act as indicators for monitoring the cluster solutions. For more in-depth information on real-time detection of changes in clusters using density-based algorithms, interested readers can refer to articles [16–18]

## 3 Methodology

This paper aims to demonstrate the influence of variability in cluster sizes on clusters’ temporal evolution. Additionally, it seeks to identify the minimum sample size required in dynamic streams. For this purpose, we implement the MONIC framework in R-software, which can be downloaded from the URL https://CRAN.R-project.org/package=clusTransition [19, 20]. A comprehensive literature review reveals the introduction of several algorithms designed for monitoring the changes in cluster solutions of streaming datasets. However, to our knowledge, no research work has been conducted to examine the effect of variability in cluster sizes on their evolution in streams. Consequently, there was no guidance available to choose the performance measures and assess how cluster size influences the changes adopted by the clusters over time.

### 3.1 Methods

In supervised learning, the performance of the model is evaluated by comparing the predicted class with the true class labels of the outcome attribute. However, in unsupervised learning problems, the true class labels are not provided to the learning algorithms. Hence, assessing the performance of unsupervised learning algorithms is quite challenging [21, 22]. Given the lack of pertinent literature, identifying performance indicators in this research study proved to be a formidable challenge. Generally, the consistency of cluster solutions over time and the accurate identification of changes in the stream represent critical performance indicators. In this study, we applied both the conventional logistic regression and the generalized additive logistic regression models to fit the binary response variable. The logistic regression model utilizes the logistic function to model the probability of a specific event. In this research, we consider survival of the smallest cluster as event of interest and record the dichotomous response variable *y*_*i*_ used in these models as:

$$\begin{array}{c} y_{i}=\{\begin{array}{cc} 1, & If\;the\;smallest\;cluster\;survive(with\;probability\;\pi (x))\\ 0, & If\;the\;smallest\;cluster\;experience\;transition(with\;probability\;1-\pi (x)) \end{array} \end{array}$$ (2)

It is computed as follows: In each iteration of the simulation, the datasets generated at time point *t*_2_ have identical cluster centers to the those that evolved at time point *t*_1_. Ideally, all the clusters at time point *t*_2_ should survive, and therefore, the small cluster in the stream should not experience any external transitions. The response variable records the status of the smallest cluster, indicating whether it survives or undergoes a transition. To model this phenomenon, the conventional logistic regression model is given as:

$$\begin{array}{c} Model\;1:logit(\pi_{i}(x))=\alpha \;+\;\sum_{j=1}^{p}\beta_{j}(x_{j}) \end{array}$$ (3)

where *x*_*j*_’s represents the co-variates included in the study and *β*_*j*_’s are the corresponding regression coefficients. The predictors and their respective levels included in the study are presented in Table 1. These predictors encompass the size of the smaller cluster, the number of clusters, the number of variables, and the separation between clusters. The model includes both their main effects and second-order interaction terms. The term *π*(*x*) = *P*(*y*_*i*_ = 1|*X*) computes the conditional probability that the smallest cluster survives at subsequent time point. We can write the binary generalised additive model structure as:

$$\begin{array}{c} Model\;2:logit(\pi_{i}(x))=\alpha \;+\;S_{1}(Size)\;+\;\sum_{j=2}^{p}S_{j}(x_{j}) \end{array}$$ (4)

**Table 1:** Overview of the factors used in simulation study.

Factors	Levels
Number of variables (d)	2, 3, 4, 5, 10, 15
Size of small cluster (*n*_1_)	10*d*k, 20*d*k, 30*d*k, 40*d*k, 50*d*k
	60*d*k, 70*d*k, 80*d*k, 90*d*k, 100*d*k
Number of clusters (k)	3, 4, 5
Time points	2
Separation value	-0.1, 0.0, 0.1
Total number of datasets	6*10*3*2*3 = 1080

The larger clusters in the datasets consist of 5000 observations each. The datasets were simulated at two time points and the changes in cluster solutions were monitored.

The terms *S*_1_ is the smooth non-parametric basis function that transform *Size* with the specific form depending on the chosen basis. On the other hand, *S*_*j*_’s are the linear functions capturing the effects of other co-variates.

Generalised Additive Models (GAMs), introduced by Hastie and Tibshirani [23], belong to a family of statistical models that utilize smoothing functions to capture the non-linear relationships between the response variable and predictors, thereby accommodating the data’s complexities. GAMs offer a reliable approach for smoothly fitting unseen data while avoiding excessive model complexity. The core concept involves fitting smooth non-linear functions to a set of predictors *X*_*i*_ to uncover the relationships among the variables in the model [24, 25]. In the case of GAMs, the relationship between individual predictors and the response variable follows some linear or non-linear smooth pattern. This pattern can add up to predict the expected value of the dependent variable.

### 3.2 Data generation

Clustering analysis is an unsupervised learning technique that seeks interesting patterns in datasets without pre-existing true class labels. Since the true class labels are unknown, and hence the true structure can not be predicted in the dataset [26, 27]. To overcome this limitation, we use the simulated datasets to achieve the study objectives. One significant advantage of using a simulation study over real-life datasets is the availability of true class labels in simulated data. This enables the tracking of data item migration between clusters based on their actual class memberships, facilitating monitoring of the cluster evolution.

### 3.3 Clustering algorithm

The MONIC framework operates under the assumption that each data item is exclusively assigned to one and only one cluster. This assumption effectively eliminates the feasibility of applying density-based and model-based clustering algorithms. This constraint leads us to focus on partitioning methods for clustering. Among these methods, one of the most common and suitable choices is the traditional *k*-means algorithm. The true number of classes (i.e., number of clusters generated in each simulation) was used as a relevant value of *k*. The *k*-means [28, 29] is one of the most widely used learning algorithms for partitioning data into a specified number of clusters *k*. Here, *k* represents the optimal number of clusters pre-specified by the analyst. The algorithm optimizes two competing criteria: objects belonging to the same cluster display high intra-class similarity, while those from distinct clusters exhibit low inter-class similarity.

## 4 Results and discussion

The results section is organized as follows: Section 4.1 presents a preliminary analysis mapping the impact of cluster size variability on the evolution of clusters in a data stream. Ten streams were specifically created for this purpose, with data items emerging at two successive time points in each stream. At time point *t*_1_ in each stream, the dataset comprised four distinct clusters with varying sizes (i.e. one small and three large clusters). Each of the larger clusters contained 5000 observations, while the size of small clusters in the streams were 50, 100, 150, 200, 250, 300, 350, 400, 450, and 500 respectively. At time point *t*_2_, four clusters with cluster centers similar to those that emerged at *t*_1_ were simulated, each containing 2,000 observations. The consistency and stability of the clusters in the second clustering are assessed, as the clusters at subsequent time periods have similar centers.

In section 4.2, data streams were generated at two consecutive time points. To ensure reliable recommendations different circumstances are encountered in artificial datasets, including 1) number of variables (d), 2) sample sizes, 3) number of clusters (*k)*, 4) and separation value between neighboring clusters. The datasets were generated using *genRandomClust()* functions from *clusterGeneration* and *cluster.Gen()* function from *clusterSim* package in R [19, 30, 31].

The number of respondents in the small cluster was determined based on the recommendations of Qiu and Joe [32]. Their recommendations indicate a linear dependence of sample size on the number of variables (d) and number of clusters (*k*). These two covariates can significantly influence the outcomes and behavior of unsupervised learning algorithms. The number of variables directly affects the complexity of analysis, and computation time. The number of clusters, on the other hand, directly determines the granularity of grouping within the data. A smaller *k* may result in clusters that are too generalized, while a larger *k* can lead to fine-grained clustering that might not capture meaningful patterns. This results in 10*d*k, 20*d*k, 30*d*k, 40*d*k, 50*d*k, 60*d*k, 70*d*k, 80*d*k, 90*d*k, and 100*d*k objects simulated across each data scenario. This recommendation also serves to justify the utilization of simulated datasets in unsupervised learning, as the actual number of clusters remains unknown in real datasets. Table 1 below present an overview of the factors included in the study.

### 4.1 Preliminary analysis

**Fig 1:**
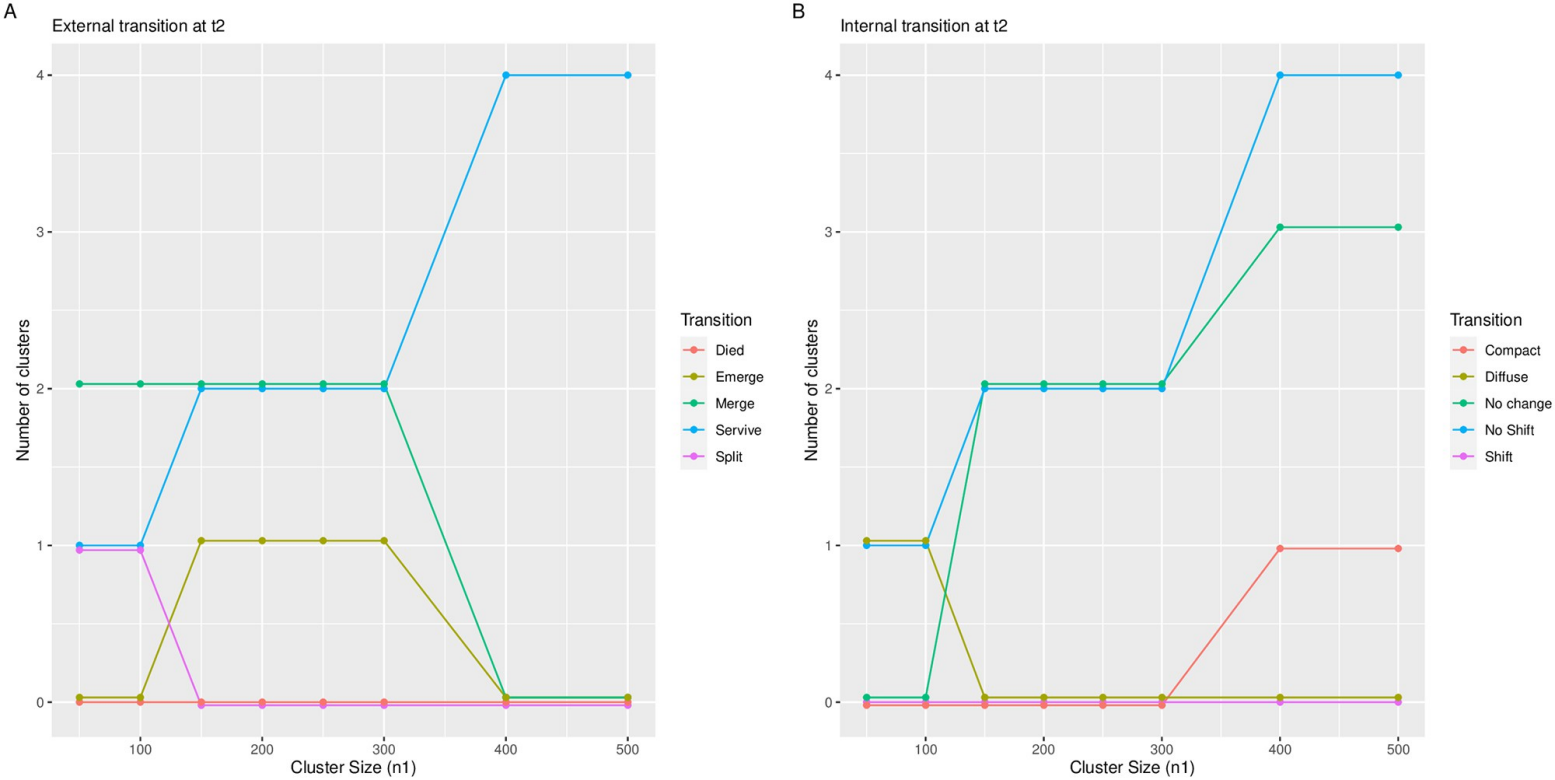
The changes adopted by clusters at time point *t*_2_ with respect to the size of the smaller cluster. The x-axis represents the smaller cluster size, while the y-axis denotes the number of clusters experiencing the corresponding transition.

Fig 1 below demonstrates the external and internal transitions adopted by the clusters at time point *t*_2_ in relation to different sizes of the small cluster. Though the datasets at time point *t*_2_ being simulated with centers identical to the clusters evolved at *t*_1_. Yet, extremely unstable clustering solutions were obtained at succeeding time points due to variability in the cluster sizes.

Sub-plot A in Fig 1 illustrates that the algorithm identified one survived, two merged, and one split candidate at time point *t*_2_. However, as the size of the smaller cluster exceeded 150 observations, two survived, two merged, and one newly emerged candidate were detected. Moreover, once the size of the smaller cluster reached 400 observations, all four clusters survived at the subsequent time point. Similarly, sub-plot B in Fig 1 depicts the internal transition of the surviving clusters at time point *t*_2_. It can be observed that the survived cluster is more diffused than its ancestor when the size of the smaller cluster is 100 observations or less. On the other hand, when the size of the smallest cluster exceeds 400 observations, one cluster experiences a change in cohesion, becoming more compact than its previous state.

**Fig 2:**
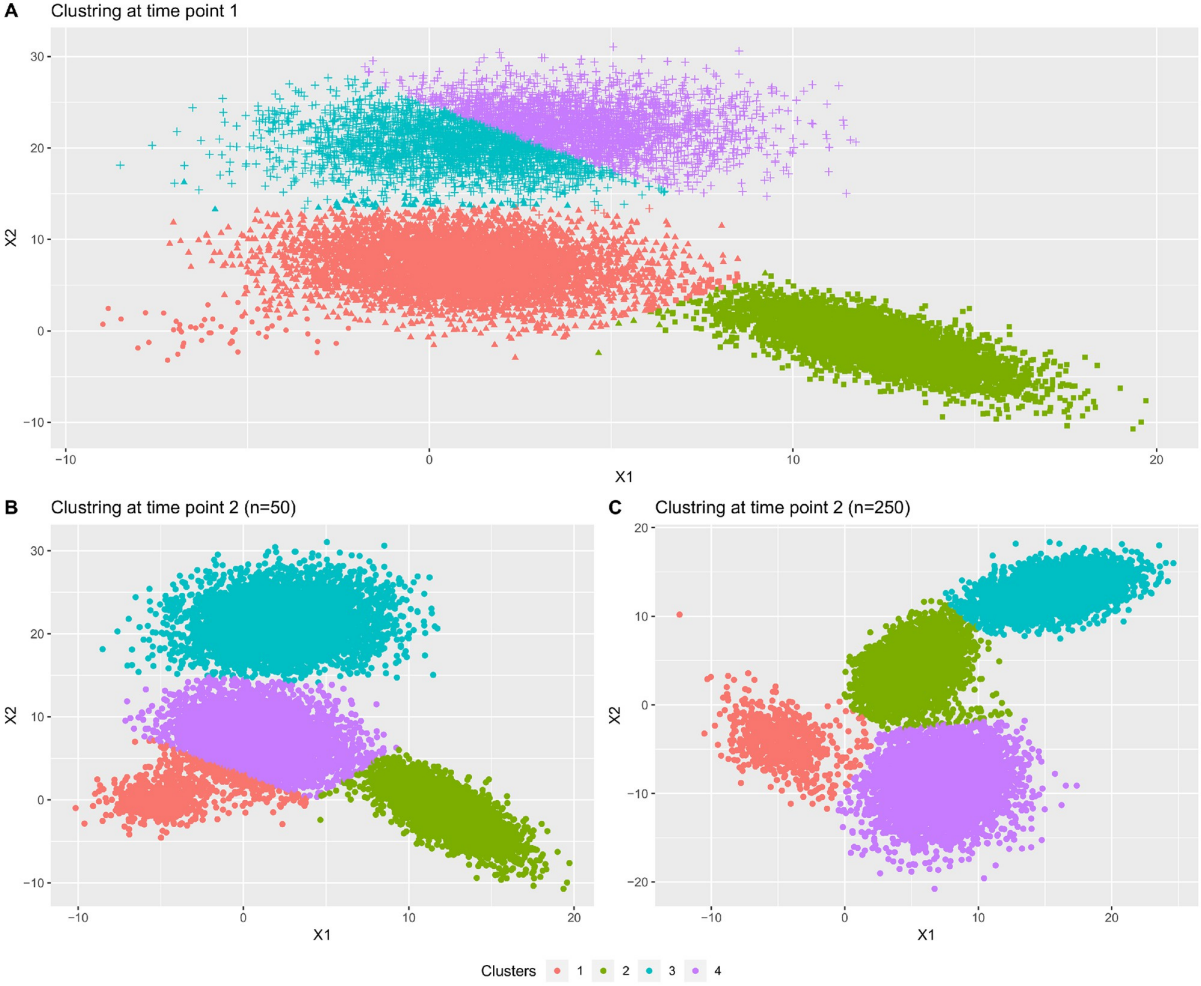
Clustering solutions at successive time points. Sub-plot A represents cluster solution at time point *t*_1_. The sub-plot B represent cluster solution at time point *t*_2_ when the size of smaller cluster is 50, whereas sub-plot C represent cluster solution at time point *t*_3_ when size of smaller cluster is 250.

This unstable clustering solution is explained in Fig 2, highlighting the impact of cluster size variability in the stream. The color scheme represents the predicted class, whereas the symbols depict the true class labels in the stream. The clustering solution at time point *t*_1_ clearly shows that the smallest class (⋅) in the dataset is a part of its neighboring larger cluster. However, with the introduction of new data points at time point *t*_2_, the cluster splits into two daughter clusters. Meanwhile, class (+) was divided into two clusters, which then merge into a single cluster at the subsequent time. The method accurately identifies one split and two merged clusters at time point *t*_2_ due to this phenomenon. However, because the smallest cluster contains more than 150 observations, it is recognised as a recently emerging candidate. Consequently, the adjacent cluster undergoes changes in cohesiveness and location. Furthermore, as the smallest segment comprises over 400 observations, all clusters manage to survive. In light of this fact, even the smallest class is now recognised as a separate cluster.

### 4.2 Model 1: Logistic modeling

To further investigate the impact of sample size on the survival of clusters over time in streaming datasets, we analyzed simulated data using logistic regression models. According to Table 2 in the usual logistic regression model, the coefficients of eight factors (Sample size, d.3, d.4, d.5, d.10, d.15, k.5, and Separation.0.1) are found to be statistically significant. Whereas six two factor interactions (d.3:Separation.0.1, d.15:Separation.0.1, k.5:Separation.0.1, d.5:k.5, d.10:k.5, and d.15:k.5) are found significant at 1% level of significance (see details in Table 2).

**Table 2:** Summary of regular logistic and GAM logistic regression models.

Factors	Model 1			Model 2		
	Estimate	Std. Error	Pr(>|*z*|)	Estimate	Std. Error	Pr(>|*z*|)
(Intercept)	-38.17095	5.76496	3.56e-11 ***	-49.12996	15.85510	0.001944 **
Sample size	0.34731	0.04949	2.25e-12 ***	-	-	
d.3	7.97918	2.68648	0.002977 **	1.14972	2.59455	0.657674
d.4	12.25383	2.97923	3.90e-05 ***	-1.0024	3.67016	0.784747
d.5	13.42242	3.05780	1.14e-05 ***	-4.3094	4.86085	0.375317
d.10	18.80360	3.5697	1.38e-07 ***	-2.9172	6.22457	0.639307
d.15	21.87555	3.8768	1.68e-08 ***	0.00000	0.00000	NA
k.4	4.29816	2.20465	0.051225	-3.3750	1.90011	0.075693
k.5	2.84153	2.77007	3.56e-06 ***	0.00000	0.00000	NA
Separation.0	2.66206	2.18551	0.223205	2.61664	2.35764	0.267061
Separation.0.1	10.8364	2.57768	2.62e-05 ***	7.01857	2.7747	0.000424***
d.3:Separation.0	-5.14193	2.45350	0.036104*	-4.18314	2.45955	0.088985
d.4:Separation.0	-2.85970	2.36917	0.227415	-2.27818	2.47028	0.356407
d.5:Separation.0	-2.84162	2.38619	0.233710	-2.21194	2.53206	0.382350
d.10:Separation.0	-1.66673	2.37178	0.482222	-0.45207	3.03939	0.881761
d.15:Separation.0	-1.66430	2.36573	0.481742	6.35778	8.87911	0.473968
d.3:Separation.0.1	-6.35238	2.45449	0.009652 **	-4.45037	2.62213	0.089652
d.4:Separation.0.1	-4.08594	2.40381	0.089174	1.25781	3.61987	0.728235
d.5:Separation.0.1	-4.03048	2.37642	0.089881	10.53441	13.7974	0.445163
d.10:Separation.0.1	-4.10004	2.40582	0.088341	17.28238	13.2744	0.192942
d.15:Separation.0.1	-6.35268	2.45433	0.009644 **	1.61724	3.13508	0.605957
k.4:Separation.0	0.87142	1.60454	0.587062	0.34037	1.83269	0.852662
k.5:Separation.0	-0.30521	1.60208	0.848913	-1.41535	2.02644	0.48490
k.4:Separation.0.1	-1.43624	1.59846	0.368912	3.58627	2.72671	0.188431
k.5:Separation.0.1	-4.94125	1.73175	0.004326 **	-5.0120	2.39089	0.036054
d.3:k.4	1.69185	2.38524	0.478141	1.39536	2.27034	0.538817
d.4:k.4	0.51388	2.37306	0.828560	3.56847	2.96395	0.228606
d.5:k.4	0.48054	2.34861	0.837879	4.45265	2.99951	0.137687
d.10:k.4	-0.63694	2.35253	0.786586	16.7661	11.3012	0.137923
d.15:k.4	-1.78648	2.36330	0.449695	15.40508	13.82757	0.265243
d.3:k.5	0.46307	2.38456	0.846023	2.84301	2.69342	0.291180
d.4:k.5	-4.13632	2.43520	0.089403	-0.06837	3.17933	0.982843
d.5:k.5	-7.67137	2.57211	0.002859 **	-4.97685	2.79758	0.075242
d.10:k.5	-8.76143	2.63695	0.000892 ***	0.34106	33.98105	0.991992
d.15:k.5	-9.96151	2.72808	0.000261 ***	-2.02651	9.06057	0.823020
Smooth term	-	-	-	edf	Ref.df	p-value
Sample size	-	-	-	6.43	6.855	7.16e-05 ***

Subsequently, we enhance our model to GAM by incorporating smooth functions of the explanatory variables. According to Table 2 all two factor interaction terms are found to be insignificant at 1% level of significance (*P* − *value* > 0.01). However, the Separation.0.1 is the only significant factor (*P* − *value* <.01) in the GAM model. The *edf* of smoothing term for sample size is 6.43 (*P* − *value* < 7.1*e* − 05) indicating that the effect of sample size on log odds is not linear.

#### 4.2.1 Model comparison

Now, let’s compare our regular logistic model with GAM logistic model using multiple performance standards. Table 3 below demonstrates some of these performance standards for model comparisons.

**Table 3:** Model selection standards.

Models	AIC	BIC	Pseudo *R*^2^
Model 1	169.9385	320.1434	81.3
Model 2	145.3006	318.8073	94.3

Comparing these standards indicates that it is now safe to conclude that the GAM performs considerably better than the regular logistic model. To further validate this comparison, we conducted the standard test using R-software’s *anova* function to statistically compare the two models. The results of the analysis of deviance for model comparison are presented in Table 4.

**Table 4:** Analysis of deviance table.

Models	Resid. Df	Resid. Dev	Df	Deviance	Pr(>Ch5)
Model 1	505.00	99.938	-	-	-
Model 2	499.57	64.441	5.4296	35.497	1.912e-06 ***

Now, with additional statistical evidence, we can confidently propose that incorporating the non-linear relationships of the covariates significantly enhances the performance of the model.

#### 4.2.2 Interpretation

**Fig 3:**
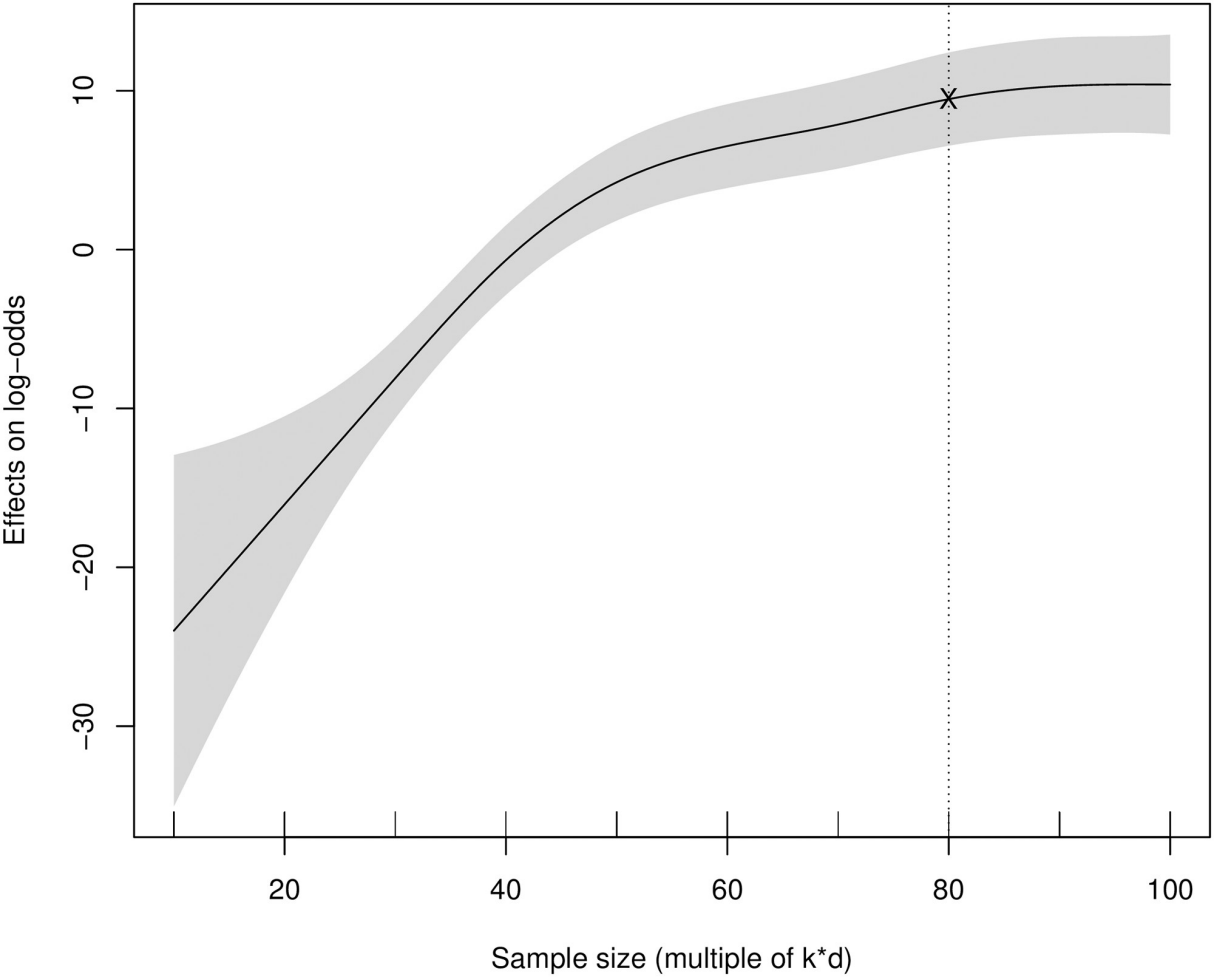
Plot from a GAM model in which the survival of smaller cluster has been modeled as a smooth function of the sample size of smaller segment for aggregate data over all levels of covariates. The standard error limits are shaded.

A comprehensive analysis of the results reveals that variability in cluster sizes has a considerable impact on the evolution of clusters. To provide adequate recommendations regarding minimum size required for detecting the correct transitions, smooth functions are plotted. Fig 3 below represents the smooth function of sample size on the log-odds of the dependent variable, as explained in Eq 2, considering all levels of the covariates. The impact of an increased sample size on the accurate detection of the transition is highly significant. The curve demonstrates a sharp ascent initially, followed by a gradual flattening once the size of the smallest class exceeds 70. In summary, for correct detection of transitions in aggregate settings, the recommended sample size is determined to be 70 * k * d.

**Fig 4:**
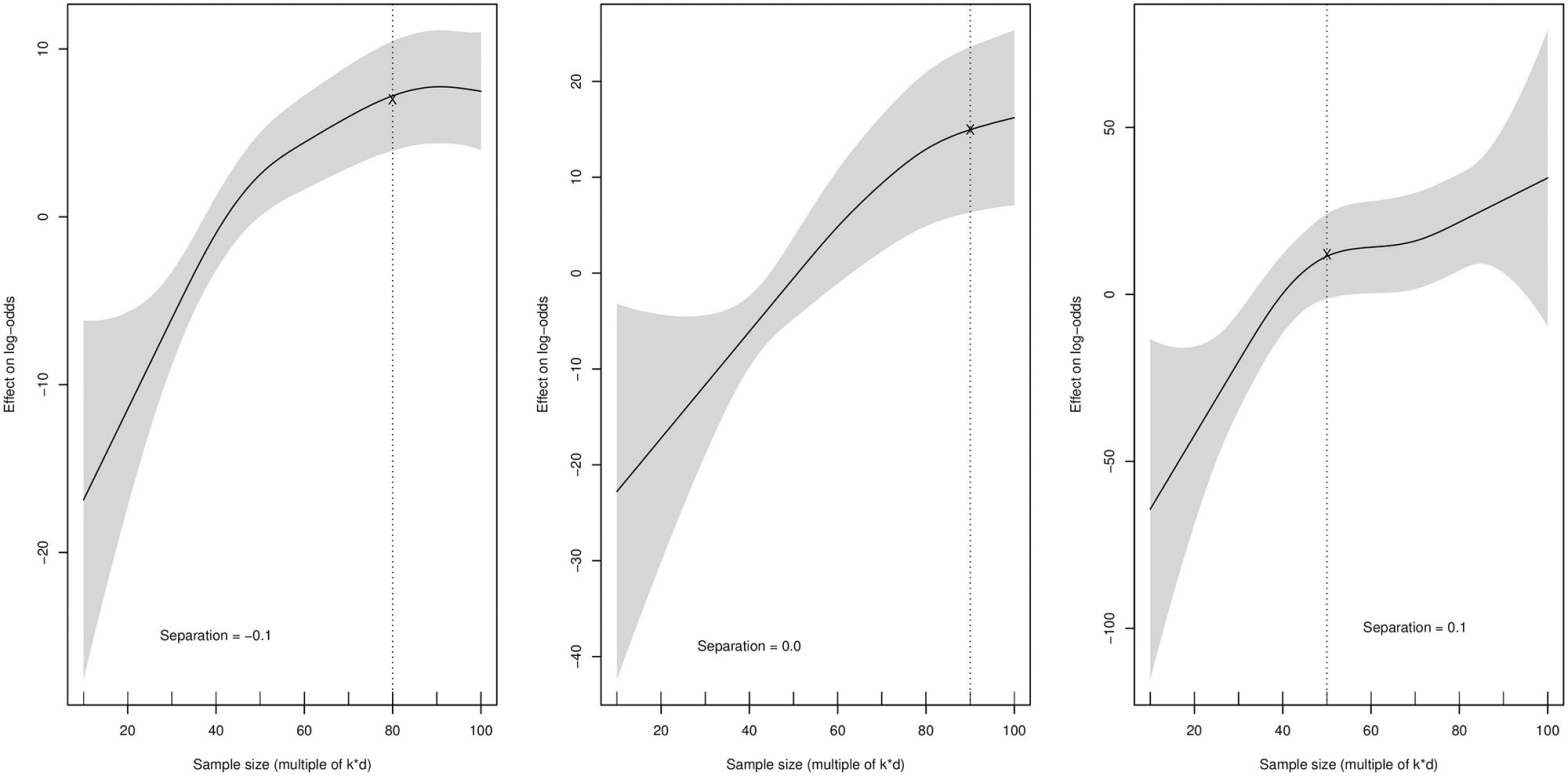
Plot from a GAM model in which the survival of smaller cluster has been modeled as a smooth function of the sample size of smaller segment for specific values of the Separation between neighboring clusters. The adequate sample size is mentioned with dashed lines.

However, Separation.0.1 is also significant in the GAM model, which suggests that separation between neighboring clusters also plays a vital role. Fig 4 below demonstrates the smooth function of sample size and its effect on the log-odds of dependent variable for different values of separation. Clearly, if there is no obvious structure in the dataset, i.e., if neighboring clusters are close to one another, then a higher sample size is required to achieve significantly improved results. In such cases, the impact of increasing the sample size of the smallest cluster is particularly strong. Thus, if there is no obvious structure, higher sample sizes are required. On the other hand, if clusters are well separated from each other, i.e., Separation.0.1, then the function is almost horizontal. This suggests that there is no additional effect of sample size once the size of the smallest cluster exceeds 50*k*d. The smooth function of sample size on the log-odds of the dependent variable explained in Eq 2 over all levels of the covariates are provided in S1, S2 Figs and S1 Text available in supporting information.

Clustering methods heavily rely on the sizes of the actual classes in a dataset. Therefore, finding the appropriate structure in datasets becomes quite tricky if the cluster size are inadequate. The issue of determining minimum sample sizes required for the smallest cluster in a dataset has been explored by a limited number of studies. For example, Dolnicar et al. [33] performed a study using simulation techniques. They put forward a suggestion regarding the smallest sample size required for data-driven market segmentation in order to precisely detect clusters. This also plays a role in the transitions of clusters when clustering time-stamped datasets. If the clustering algorithm fails to capture the underlying pattern accurately, it can lead to highly unstable cluster outcomes at consecutive time points.

## 5 Conclusion

Clustering is a data organization technique that groups data points into clusters. The key characteristic is that points within the cluster are more similar to each other than to those in different clusters. However, in recent times, a substantial volume of data is generated in the form of continuous streams. Consequently, the underlying structure of data evolves over time, leading to the development of algorithms specifically designed to monitor and adapt to these changes. In recent decades, researchers have focused on tracking changes in cluster solutions for temporal datasets. However, to the best of our knowledge, no study has been conducted to explore how variations in cluster sizes affect the temporal evolution of clusters. The primary objective of this study is to determine the minimum sample size required for effectively monitoring changes in temporal datasets. Results of study reveal that increasing size of the smallest cluster within the dataset significantly improves the outcomes. Especially in situations where no distinct segmentation structure exists, larger sample sizes are required for precise detection of the changes. The findings suggest that at least 70*k*d observations are required in the smallest cluster. This implies that, for accurately detecting transitions in temporal datasets, a sample size equal to 70 times number of variables and number of clusters is deemed sufficient. However, in cases where the dataset lacks clear segmentation, significantly higher sample sizes are necessary. These findings are substantial because tracing and monitoring the changes in clustering solutions have a wide range of applications in every field of research.

## 6 Limitations and future work

The MONIC framework operates under the assumption that each individual object is exclusively allocated to a single cluster. This assumption restricts the study to only partitioning clustering algorithms, effectively ruling out the possibility of applying density-based and model-based clustering algorithms. In the future, this study could be expanded to encompass density-based algorithms.

## Supporting information

S1 Text Smooth function of sample size. (TXT)

S1 Fig Smoothing curve from GAM model in which the survival of smaller cluster has been modeled as a smooth function of the sample size. (TIF)

S2 Fig Smoothing curve from GAM model in which the survival of smaller cluster has been modeled as a smooth function of the sample size. (TIF)

S1 Data (XLSX)
