# Supplementary figures and images for: The least sample size essential for detecting changes in clustering solutions of streaming datasets
Source: PLoS One. 2024 Feb 20;19(2):e0297355. doi: 10.1371/journal.pone.0297355 (PMC10878511; doi:10.1371/journal.pone.0297355)

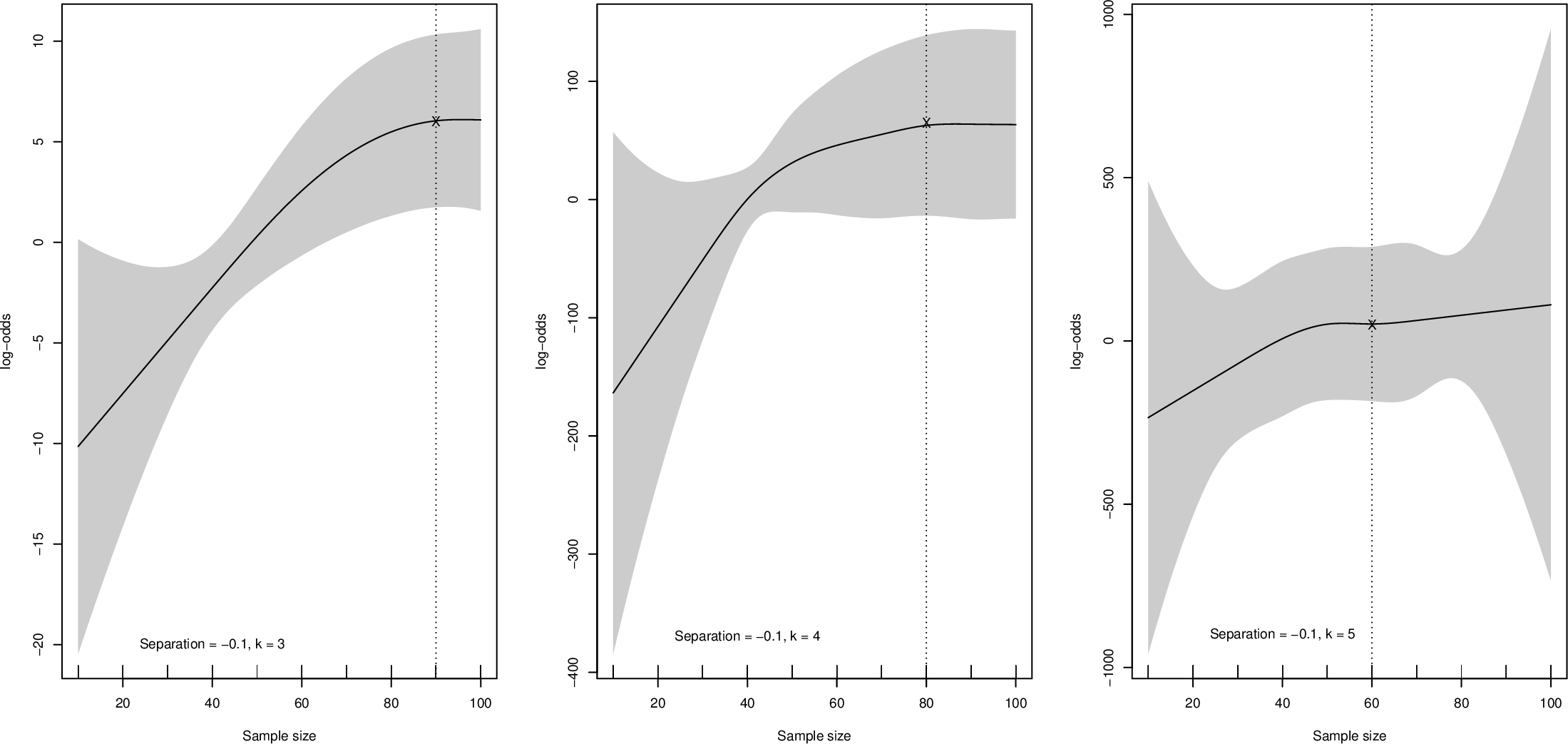

Supplement: S1 Fig — (TIF) [file pone.0297355.s002.tif]

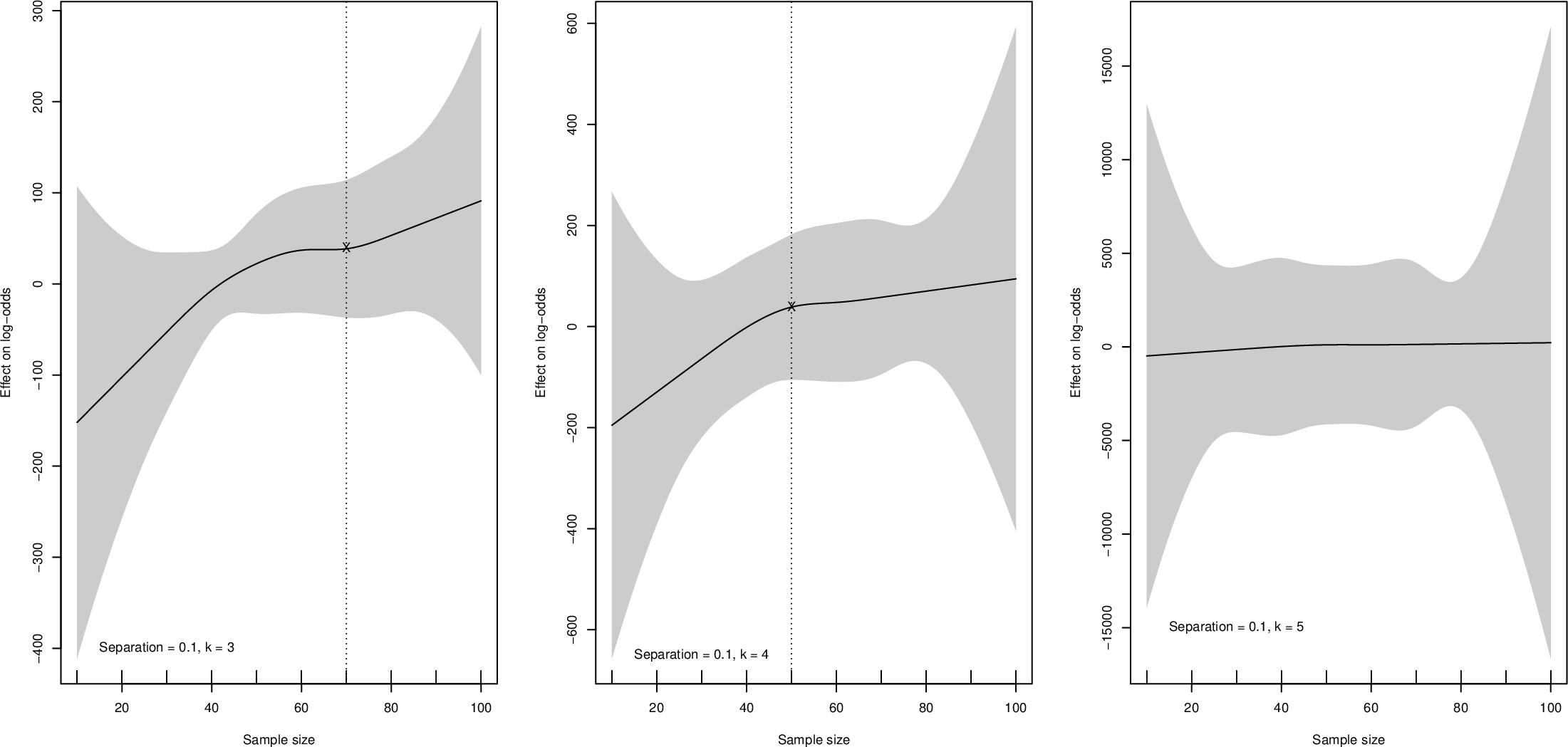

Supplement: S2 Fig — (TIF) [file pone.0297355.s003.tif]
